# Book review: Treatment and recovery of eating disorders – edited by Daniel Stein and Yael Latzer

**DOI:** 10.1186/2050-2974-2-4

**Published:** 2014-01-31

**Authors:** Lisa Dawson

**Affiliations:** 1 School of Psychology, University of Sydney, Sydney, Australia

**Keywords:** Book review, Treatment, Recovery, Eating disorders

## 

This book aims to provide a current review of established treatment options and prognostic issues in eating disorders. This is a multi-authored, easy-to-read book with contributions from leading international researchers. The book provides a broad approach to eating disorders with chapters focussing on anorexia nervosa (AN), bulimia nervosa (BN), binge eating disorder, and obesity. Chapters focus on a number of different models and approaches in the treatment and conceptualisation of eating disorders such as cognitive behavioural therapy (CBT), psychodynamic psychotherapy, family therapy, and pharmacotherapy. Multiple perspectives on eating disorders are provided, with chapters written by dieticians, psychologists, psychiatrists and family therapists, reflecting the multidimensional nature of eating disorders as well as the multidisciplinary approach often involved and required for treatment. The first half summarises the knowledge base in regards to the most significant treatment strategies in eating disorders. The last four chapters deal with issues related to outcome and prognosis.

This book opens with an informative overview of the latest data on the most efficacious treatment options in eating disorders. The available evidence for CBT, self-psychology, family-based treatment interventions and medications is reviewed, providing a useful summary of the current evidence base and setting the scene for the rest of the book. Following this, the second chapter focuses on psychodynamic approaches to eating disorders with Banker providing an enlightening review of the history of psychoanalysis and how such treatments have been applied to the eating disorders. In chapter three a thorough overview of the current evidence-base for treatment of BN is provided, including a review of the evidence for established and less established psychotherapies, self-help approaches, and pharmacotherapy. The authors have extensively reviewed the literature providing a useful summary for those new to treating BN or those wanting an update on current treatment approaches.

In chapter four Karwautz, Huemer and Wagner offer an extensive overview of psychopharmacological treatments. The evidence ratings for different medications for the eating disorders are comprehensively documented. The authors provide user-friendly tables summarising the results of studies using antipsychotic agents, selective serotonin releasing inhibitors (SSRIs), antidepressants other than SSRIs, anticonvulsants, and other agents. Conclusions are drawn using the National Institute of Clinical Excellence (NICE) criteria of evidence as guidelines making this chapter of use for those wanting an accessible summary of psychopharmacological treatment recommendations.

Moving on to obesity, the metabolic, lifestyle and psychological features associated with reduced-weight former obese individuals are reviewed in chapter six. The authors, Margalit and Berry, outline important and revealing data that suggest that there are significant differences between those who have successfully lost weight after a history of obesity and those who have never been obese, despite having similar Body Mass Indexes. Such differences interestingly include metabolism changes as well as different psychological and lifestyle needs in order to remain weight stable. Maintaining this kind of weight loss, the authors highlight, involves a three-fold approach and lifetime commitment of restricted eating, strenuous physical activity, and frequent self-monitoring. This topical chapter raises difficult and thought-provoking questions as to the helpfulness of encouraging weight loss, given so many individuals might enter weight cycling.

In chapter six, Golan provides insight into the day-to-day practice of dieticians working in eating disorder treatment and effectively draws attention to the importance of the dietician’s role in the treatment team. Tobin then provides a well-considered rationale for using psychodynamic approaches with eating disorder patients, offering evidence from randomised controlled trials as well as clinical practice. This chapter describes and advocates developing an evidenced-based approach that combines both cognitive behavioural and psychodynamic approaches.

An informative review of the most well tested CBTs for eating disorders, including BN, AN, and overweight patients is then presented by Hay and Touyz. The theoretical background as well as treatment principles are discussed and the transdiagnostic approach used in eating disorders is described. The phases of the treatments are helpfully reviewed offering a practical insight into the nature of treatment and the authors provide useful suggestions for further reading.

Issues pertaining to therapists, specifically the supervision of clinicians and managing counter-transference, are then reflected upon. In chapter nine Mitrany discusses the art of supervision in the treatment of eating disorders, focussing on both the supervision of case management for multidisciplinary professionals and the supervision of psychodynamic psychotherapy. The importance of professionals being on the same page is highlighted and valuable and practical case vignettes are provided to illustrate important points. Golan and colleagues then discuss the challenge of counter transference and outline how it can manifest when working with eating disorder patients and the effects of this on team dynamics and treatment outcome. Coping approaches to manage counter-transference are reviewed, providing practical tips for facilitating effective treatment processes for both the patient and the therapist.

Family-based approaches are also considered with multi-family therapy and new applications for FBT discussed. Depestele and Vandereycken firstly consider an inpatient multi-family therapy approach for adolescents with eating disorders, describing the goals and methods of multi-family groups. They provide session-by-session overviews with effective, illustrative vignettes and evaluate the benefits of such an approach. Next, Loeb and Le Grange outline new and innovative applications of FBT. This includes applying FBT for people with BN, young adults with AN (e.g., ages 18 – 25), for those with subclinical AN, and for overweight children. New and exciting research that is taking place in this area is reported.

The last four chapters of the book change focus from treatment to concentrate on outcome and recovery. Chapter 13 explores the patient’s perspective of recovery from AN. The authors present a study of recovered women interviewed about the meaning of their recoveries and offer rich insights into the experience and significance of this process. A model of the recovery process from the patient’s perspective is presented, offering a strong appreciation for the multidimensional nature of recovery. Chapter 14 investigates the difficulties in studying recovery from AN due to the lack of consensus as to how recovery should best be defined. The authors present a thorough review of the literature, identifying at least ten definitions used in the field. They highlight the clinical and research implications of this lack of consistency and provide suggestions for future research. The importance of establishing a consensus definition is discussed, raising important questions for the field.

The final two chapters of the book focus on recovery from BN. Firstly, a qualitative study exploring how women who have recovered from BN describe their recovery process is presented, raising powerful and resonating insights into the BN patient’s perspective. Lastly, recovery from BN is comprehensively explored by Hay with an emphasis on defining recovery from BN, naturalistic outcome, and outcomes with treatment.

A valuable book for practitioners, researchers, and students, this book provides a broad, evidenced-based framework of the treatment of eating disorders, covering an impressive range of topics. Detailed reviews of the research are presented offering an up-to-date summary of current thinking in eating disorder treatment across diagnoses. I found this book to be informative and thought-provoking. A particular strength is the comprehensive and holistic approach that has been taken in which multiple perspectives are presented. With an emphasis on empirical evidence, the book encapsulates the varying approaches to the treatment and conceptualisation of eating disorders. The inclusion of chapters on recovery and outcome is also significant as this is an important yet generally less discussed area. Similarly, despite being an important source of knowledge, patient perspectives are not often included in such texts. This addition gives richness to the book and offers insight into an important area with which researchers and clinicians need to be familiar.

## Competing interests

The authors declare that they have no competing interests.

